# Clustering-cum-regression based model and performance analysis for early prediction of heart disease

**DOI:** 10.1038/s41598-026-40626-z

**Published:** 2026-02-18

**Authors:** Manoj Tolani, Yazeed AlZahrani, Gaurav Suman, Pankaj Kumar, Arun Balodi, Ambar Bajpai

**Affiliations:** 1 Department of Electronics and Communication Engineering, Jaypee Institute of Information Technology, Noida, 201309 Uttar Pradesh India; 2 Department of Computer Engineering and Information, College of Engineering in Wadi Addawasir, Prince Sattam bin Abdulaziz University, Wadi Addawasir, Saudi Arabia; 3 Manipal Institute of Technology, Manipal Academy of Higher Education, Manipal, India; 4 Department of Electronics and Communication Engineering, Dayananda Sagar University, Bengaluru, Karnataka India; 5 Department of Electrical, Electronics and Communication Engineering, GITAM University, Bengaluru, Karnataka India

**Keywords:** Wireless body area network, Prediction, Regression, Medium access control, K-Means Clustering, Biomedical engineering, Congenital heart defects

## Abstract

In real-time health monitoring systems, Wireless Body Area Networks (WBAN) are widely recognized for collecting various disease parameters using sensors. The collected data can be used for the early prediction of diseases. To address the growing need for accurate and efficient heart disease prediction, we introduce a novel hybrid approach that combines K-Means clustering with advanced regression techniques to analyze various factors in heart health monitoring. This integrated method utilizes the strengths of unsupervised and supervised learning to enhance predictive accuracy across both training and testing datasets. Our analysis focuses on 12 critical feature parameters, systematically clustered using K-Means to uncover inherent patterns and relationships. These parameters are then rigorously evaluated through multiple regression models to determine their predictive significance. By employing K-Means to assess parameter relevance within defined ranges, the proposed framework ensures robust feature selection and improved model interpretability. To validate its effectiveness, we benchmark our approach against widely used machine learning models, including Decision Tree Regression, K-Nearest Neighbor, Support Vector Machine (SVM), Kernel SVM, and others. The results demonstrate that our method not only outperforms traditional techniques but also offers a scalable and reliable solution for real-world healthcare applications. The prediction accuracy and false-prediction performance parameters were analyzed to compare the proposed method with existing heart disease prediction models. Earlier approaches reported accuracies up to 85%, with limited improvement in recall, specificity, and F1 score. In contrast, the newly proposed hybrid model–integrating Random Forest regression with K-Means clustering–achieved a significantly higher accuracy of 91%, along with improved recall (0.8864), specificity (0.9583), F1 score (0.8977), and ROC–AUC (0.9155). These quantitative performance gains, obtained without increasing model complexity, clearly demonstrate the superiority and robustness of the proposed approach over traditional prediction methods.

## Introduction

Patient health monitoring is a crucial requirement in today’s world. The researchers work in various domains to efficiently monitor patients’ health status. Due to the COVID-19 pandemic, nowadays, doctors, health practitioners, and patients prefer remote health monitoring. However, sometimes patients prefer an offline visit to the clinic instead of an online consultation. To resolve the issues and for the convenience of the patients, the researchers are working on a relevant solution. Additionally, regular patient monitoring can be a key factor in successful treatment. The average number of visits required for the patient treatment varies and depends upon the type of disease^1^.

Previous research shows that for certain diseases, regular visits to the clinic become a key factor in successful treatment, such as Type 1 Diabetes. However, due to unavailability of the doctor or due to failure of the patient to visit the clinic, the success rate of treatment reduces to 30–40%^2^. To address such issues, the researchers are working on a Wireless Body Area Network (WBAN). WBANs enable applications across safety, personalized healthcare, chronic disease monitoring, fitness tracking, maternity care, and elderly care, leveraging intelligent computing for real-time data acquisition and analysis^3^. The WBAN is a very efficient method for the real-time monitoring of the patient. In WBAN, sensors are placed on the body, and sensor nodes regularly monitor various parameters, transmitting data samples to users’ mobile devices. The user’s mobile device aggregates the data and transmits it to the health monitoring station, where health practitioners analyze the data for the early prediction of disease. The researchers are working in two fields of WBAN for efficient operation. The first requirement of the WBAN is to transmit the data in an energy-efficient manner. The researchers have proposed various energy-saving methods for bandwidth utilization to efficiently transmit data^4,5^. The researchers have introduced several medium access control protocols designed to enhance the energy efficiency of data transmission. They recommend using the IEEE 802.15.6 standard protocol suite for both the physical layer and the MAC layer in body area network (BAN) communication^6^.

The researchers are also developing efficient data analysis methods for the early detection and prediction of the disease. After the data acquisition and transmission, the received data is analyzed by the health practitioners for the early prediction of the disease. For the efficient prediction of the disease, the researchers have proposed various artificial intelligence and machine learning methods. In previously reported works, researchers have focused on various regression methods. However, it is revealed from the previous research studies that different protocols perform efficiently for different types of disease. Moreover, for a few diseases, the early prediction of the disease has been proven to be a lifesaver and a game changer in the treatment of the disease^7^.

In^8^, researchers conducted a comprehensive comparison of nine prominent clustering algorithms, assessing their performance on artificial datasets with diverse characteristics. The study also explored the sensitivity of these algorithms to various parameter configurations. Selecting the appropriate clustering or machine learning algorithm for a given dataset can be challenging. The use of artificial datasets in this work allows for extensive experimentation with an unlimited number of samples and the ability to modify dataset properties.

The heart is a vital organ that plays a crucial role in circulating blood throughout the body. The improper functioning of the heart can lead to various diseases in the body. The healthy functioning of the heart is a crucial requirement for human beings to lead long and healthy lives. Therefore, in the present work, we have analyzed the heart-related features and dataset for the early prediction of heart diseases. In previously reported works, researchers have presented various regression methods for predicting heart disease. However, it is evident that the accuracy of the existing machine learning models is low. Additionally, the researchers have not analyzed the dataset using clustering and regression methods. The clustering-based feature analysis facilitates the categorization of the dataset, providing more relevant and accurate predictions using the post-regression method. Additionally, a comparative accuracy analysis of the various prediction methods is also lacking in the previously reported works.

The novelty and the contribution of the proposed work are explained below:

- Novel introduction of a hybrid approach utilizing K-means clustering-based data analysis and regression-based algorithms applied to the specific context of Early Prediction of Heart Disease, utilizing the dataset from (Center for Machine Learning and Intelligent Systems)^9^.
- Data analysis of various attributes (feature parameters) is performed using a K-Means-based clustering algorithm (hybrid clustering-cum-regression).
- The 12 feature parameters are used for the accurate detection of heart diseases.
- The comparative analysis of various regression methods, i.e., Decision Tree Regression, K-Nearest Neighbor, Support Vector Machine, Kernel SVM, Logistic Regression, Naïve Bayesian, Random Forest Regression, has been done.
- The confusion matrix and accuracy are analyzed for the performance comparison.

## Related studies and background

As previously discussed, various research studies have been reported for the analysis of heart disease. In^7^, the authors propose a hybrid machine learning method for early heart disease prediction. The authors have compared 13 feature parameters for predicting heart disease. The paper compares the protocol’s performance with the decision tree and random forest regression methods. The proposed protocol of this paper shows the highest accuracy and lowest classification error in the results.

**Table 1:** Summary of Heart Disease Prediction Models.

Authors	Objective	Contribution	Conclusion
X. Yuan 2022^10^	To develop a prediction model for the early prediction of heart-related disease.	The authors have developed a Fuzzy-GBDT model to simplify data complexity. By employing gradient boosting decision trees, they aim to enhance the generalization capabilities of binary classification. Additionally, they have introduced a method to address overfitting.	The findings indicate that the Bagging-Fuzzy-GBDT model significantly outperforms other models in terms of both accuracy and stability, making it highly effective for both binary and multi-class classification tasks.
S. Mohan 2019^7^	The author aims to develop a model with significant features by using a Machine Learning algorithm for the early prediction of cardiovascular disease.	In order to develop the proposed prediction model, the authors have used numerous feature combinations and proven classification techniques.	By integrating a hybrid random forest with a linear model, the authors have developed a heart disease prediction model that achieves an enhanced performance, boasting an accuracy rate of 88.7%.
N L Fitriyani 2020^11^	The author aims to create an efficient heart disease prediction model that can identify and remove outliers.	The model was created using two publicly available datasets, Statlog and Cleveland. Statlog and Cleveland, two openly available datasets, were used for the development of the model. The performance of the developed model was compared with existing machine learning models and previous research findings.	Below mentioned accuracy was achieved: Statlog dataset (95.90%) and the Cleveland dataset (98.40%). These results obtained outperformed the accuracy of other models and previous research outcomes.
C. Ordonez 2016^12^	The author addressed the limitations of current algorithms by reducing the number of searches and minimizing the number of association rules in the training set.	Association rules were applied to a real dataset containing medical records of patients with heart disease. Real datasets having the medical details of patients with heart disease were used when applying association rules.	The proposed method reduced the number of association rules while achieving high prediction accuracy.
Y. Pan 2020^13^	The author aims to develop a quick diagnosis method for quick clinical tests and quick collection of individual patients’ histories.	To aid and enhance patient prognostics for cardiac disease, the EDCNN approach was created.	The test results show that in comparison with the performance of Neural Networks, the proposed model efficiently predicts the risk level.
D. Rohan et al. 2025^14^	The authors conducted an extensive analysis to identify the most effective model for heart disease prediction using AI.	A total of 11 feature selection techniques and 21 classifiers, including hybrid deep learning models, were evaluated.	Among all models, XGBoost achieved the best performance with 97% accuracy, 0.98 F1-score, and 0.98 AUC, demonstrating its superiority in heart disease prediction.

The researchers have also proposed a data analysis method for the efficient prediction of disease and more relevant results. In^15^, the authors propose a risk factor analysis for the early detection of the disease. For the risk factor analysis, the authors propose a K-Means-based data clustering approach. The authors have used eight prediction feature parameters for the data analysis. Chittampalli *et al.* have compared three regression methods for early heart disease prediction. The authors compared Random Forest, Vector Support, and Logistic Regression for performance analysis^16^.

Kavitha *et al.* proposed a hybrid regression method (decision tree cum random forest regression) for improved accuracy. The performance comparison shows higher accuracy and fewer classification errors^17^. Lakshamanarao *et al.* proposed efficient sampling for the feature selection and regression methods for the optimal performance^18^. The authors claimed higher accuracy in classification with respect to existing methods. The other machine learning algorithms and data analysis methods are proposed in^19^ and^20^. Razu *et al.* have proposed four-tier prediction schemes for heart disease^21^. In^22^, the authors introduced a fusion-based strategy for disease prediction that integrates Federated Learning with ANOVA and Chi-Square feature selection, along with Linear Discriminant Analysis for feature extraction. Their approach achieved an accuracy exceeding 88% on the Cleveland Heart Disease dataset. Similarly, in^23^, a predictive framework was proposed that combines a Modified Artificial Bee Colony (M-ABC) algorithm with k-Nearest Neighbors (KNN) to optimize feature selection, thereby improving classification accuracy for heart disease prediction using the UCI Cleveland dataset. Similarly, many other researchers have proposed AI/ML-based regression methods for the early heart disease prediction, which will be helpful in future wireless networks^9,19–21,24–32^. In^33^, the authors proposed a predictive learning model that integrates polynomial feature engineering with SMOTETomek-based data balancing, achieving an accuracy of 98.82% for heart disease prediction on the UCI dataset. The contribution of the researchers in recent years is explained below in Table 1.

We have analyzed many research works based on inclusion and exclusion criteria. We have shortlisted five research works closely related to our proposed work. In all the previous research works^10–13^, researchers have reported various methods for achieving high prediction accuracy. Some researchers have proposed hybrid models combining two different regression methods. For instance, in^34^, the authors developed a hybrid model based on a Random Forest and a Support Vector Machine. In^35^, an optimal feature selection method was proposed to enhance the performance of machine learning regression models.

To the best of our knowledge and based on the literature review conducted, it appears that while numerous studies have explored heart disease prediction using various datasets, most approaches either rely solely on clustering or regression techniques, without leveraging the combined strengths of both. The existing research on heart disease prediction predominantly employs either clustering techniques for risk factor analysis or regression-based models for classification and prediction. While several hybrid approaches have been proposed, such as combining different regression algorithms (e.g., Decision Tree with Random Forest) or integrating feature selection with predictive models, these methods do not exploit the complementary strengths of clustering and regression within a unified framework. Moreover, recent heart disease prediction methods also utilize deep learning and ensemble models to enhance accuracy; however, these approaches face challenges such as high computational costs, large data requirements, and limited interpretability. The researchers have not utilized the hybrid features of both clustering and regression methods.

Clustering is instrumental in revealing hidden patterns and grouping similar data points, which is particularly valuable in heterogeneous medical datasets where patient profiles exhibit significant variability. By leveraging K-Means clustering, we can identify coherent clusters that capture underlying health conditions and risk factors. Regression complements this process by quantifying the relationships among these features and enabling precise prediction of outcomes. The integration of clustering and regression creates a synergistic framework, first organizing the data into meaningful structures and then applying predictive modeling on these well-defined clusters, resulting in enhanced accuracy, robustness, and interpretability. The hybrid features of clustering-cum-regression provide better filtering capability and can improve the accuracy of the heart disease prediction. Therefore, in the present work, we have proposed cluster-cum-regression protocol.

## Methodology

As we previously discussed, researchers have analyzed heart-related features for the early prediction of disease. However, in the previously reported works, the researchers have not studied the relevance of the result using clustering cum regression methods. Therefore, in the proposed work, we first analyzed the dataset based on various medical features. The data analysis is done before processing the data for clustering and regression. The dataset is collected from the UCI Machine Learning Repository (Center for Machine Learning and Intelligent Systems)^9^. Despite the database containing 76 attributes, published experiments typically utilize only 14 of them. Notably, the Cleveland database has been the primary resource for machine learning researchers to date. The important parameters used for feature extraction are chest pain type, blood pressure, cholesterol, fasting blood glucose, EKG restecg, maximum heart rate, exang, ST segment depression, ST slope, vessels fluoroscopy, thallium, and number of diagnoses of heart disease (angiographic disease status). Of these 14 important feature parameters, during the analysis, it is observed that two feature parameters, i.e., Vessels Fluoro and Thallium, are patient-specific and depend upon other parameters as well. These parameters support doctors for an exact diagnosis of the heart-related problem, providing more insight into the patient under diagnosis. Hence, this work considers 12 feature parameters for further processing. Before processing the dataset, it is coded into numerical forms. The parameters that are decoded into numerical values are explained below in Table 2.

**Table 2:** Numerical decoding of heart disease dataset parameters.

**Parameter**	**Description**	**Value**	**Meaning**
**fbs**	Fasting blood sugar > 120 mg/dl	1 0	True False
**cp**	Chest pain type	1 2 3 4	Typical angina Atypical angina Non-anginal pain Asymptomatic
**restecg**	Resting ECG results	0 1 2	Normal ST-T wave abnormality Left ventricular hypertrophy
**sex**	Biological sex	1 0	Male Female
**exang**	Exercise-induced angina	1 0	Yes No
**num**	Heart disease diagnosis (angiographic)	0 1	<50% diameter narrowing >50% diameter narrowing
**slope**	Slope of peak exercise ST segment	1 2 3	Upsloping Flat Downsloping

In order to address data heterogeneity, pre-processing involves normalization and imputation of the missing values. In this work, min-max scaling is applied to map values into a [0, 1] range, thereby mitigating discrepancies in units and scales. Z-score standardization is used where variance sensitivity is critical, centering the data at a mean of zero with a variance of one. Missing values are handled through imputation, i.e., the mean or median for continuous attributes based on distribution characteristics; the median for skewed data, the mean for symmetric data, and mode-based imputation for categorical variables. These steps ensure consistent, complete, and standardized input for subsequent K-Means clustering and regression analysis. The dataset exhibited moderate class imbalance, with a higher proportion of samples representing the absence of disease compared to those indicating its presence. To address this, stratified sampling is applied during cross-validation to preserve class proportions across folds, and class-weight adjustments are used in algorithms such as SVM and Random Forest. After data pre-processing, the K-means clustering is used for the data analysis of the various parameters. Let the input dataset be defined as:

1 $$\begin{aligned} D = \{(x_i, y_i)\}_{i=1}^{n} \end{aligned}$$

Here, $x_i \in \mathbb {R}^d$ represents the feature vector of the $i^{\text {th}}$ patient, while $y_i \in \{0, 1\}$ indicates the diagnosis outcome (0 for healthy, 1 for heart disease). This formulation provides a supervised learning framework for binary classification. Clustering-based data pre-processing can be represented by below equation:

2 $$\begin{aligned} \min _{\mu _1, \dots , \mu _K} \sum _{k=1}^{K} \sum _{x_i \in C_k} \Vert x_i - \mu _k\Vert ^2 \end{aligned}$$

where, *K* denotes the number of clusters, $C_k$ represents the set of data points assigned to cluster *k* and $\mu _k$ is the centroid of the cluster *K*.

**Fig. 1:**
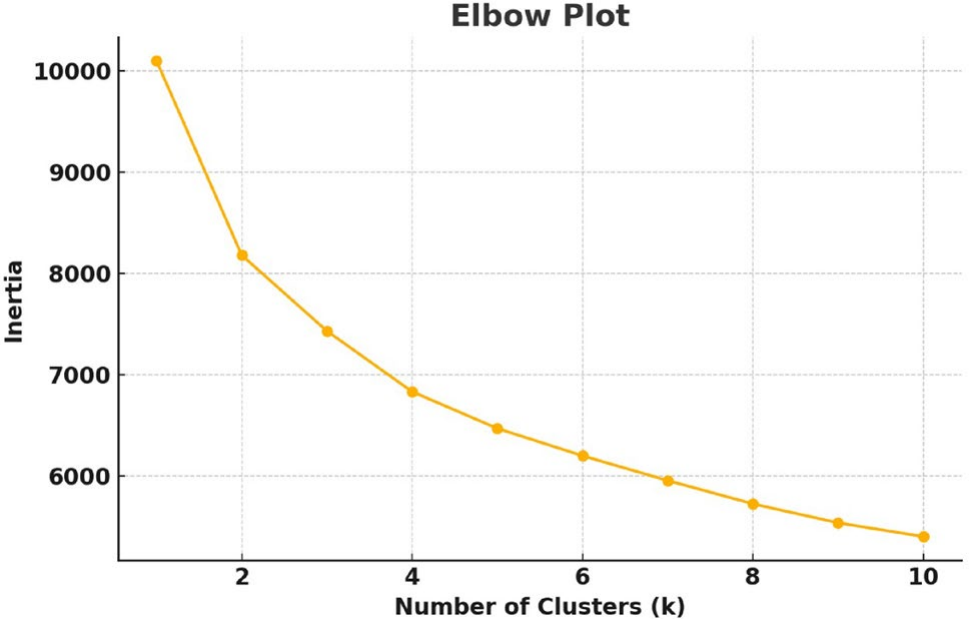
Elbow plot illustrating the optimal number of clusters for K-Means based on inertia values.

This is the objective function for K-Means clustering, which minimizes the intra-cluster variance by finding optimal centroids $\mu _k$. It groups similar data points together to improve learning efficiency during classification. Before clustering each feature, the data is first analyzed to determine the optimal number of clusters. Fig. 1 represents the elbow plot for the optimal number of clusters based on inertia values. Inertia in K-Means clustering represents the within-cluster sum of squared distances (WCSS) between each data point and its assigned cluster centroid. It measures cluster compactness, with lower values indicating tighter clusters. Clustering techniques help identify homogeneous groups within the data, group similar data points together, and detect outliers. This step helps in understanding the underlying patterns and relationships within the cluster, leading to improved accuracy of the regression algorithms. This step provides the important feature parameters that can be focused on for further analysis. In this work, the K-Means clustering technique is employed due to its simplicity, ease of implementation, and scalability, making it well-suited for large datasets.

In the current framework, decision modeling at each local node does not produce final, independent decisions. Instead, it generates decision-support features such as cluster assignments based on the identification of a single important parameter, such as age. These outputs are integrated into the global model alongside raw attributes to enhance predictive capability. Specifically, after applying K-Means clustering at each node, the cluster identifiers and associated metrics, including intra-cluster variance and distance to centroids, are treated as enriched features for the global decision layer. This hierarchical approach ensures that local clustering captures node-specific patterns and homogeneity, while the global integration enhances the observed patterns without creating conflicts between local decisions. The final decision is computed centrally using regression algorithms, selected as the best-performing among several tested on the dataset, and trained on both raw features and cluster-derived indicators. This approach improves learning efficiency and prediction accuracy by combining localized insights with global consistency.

**Fig. 2:**
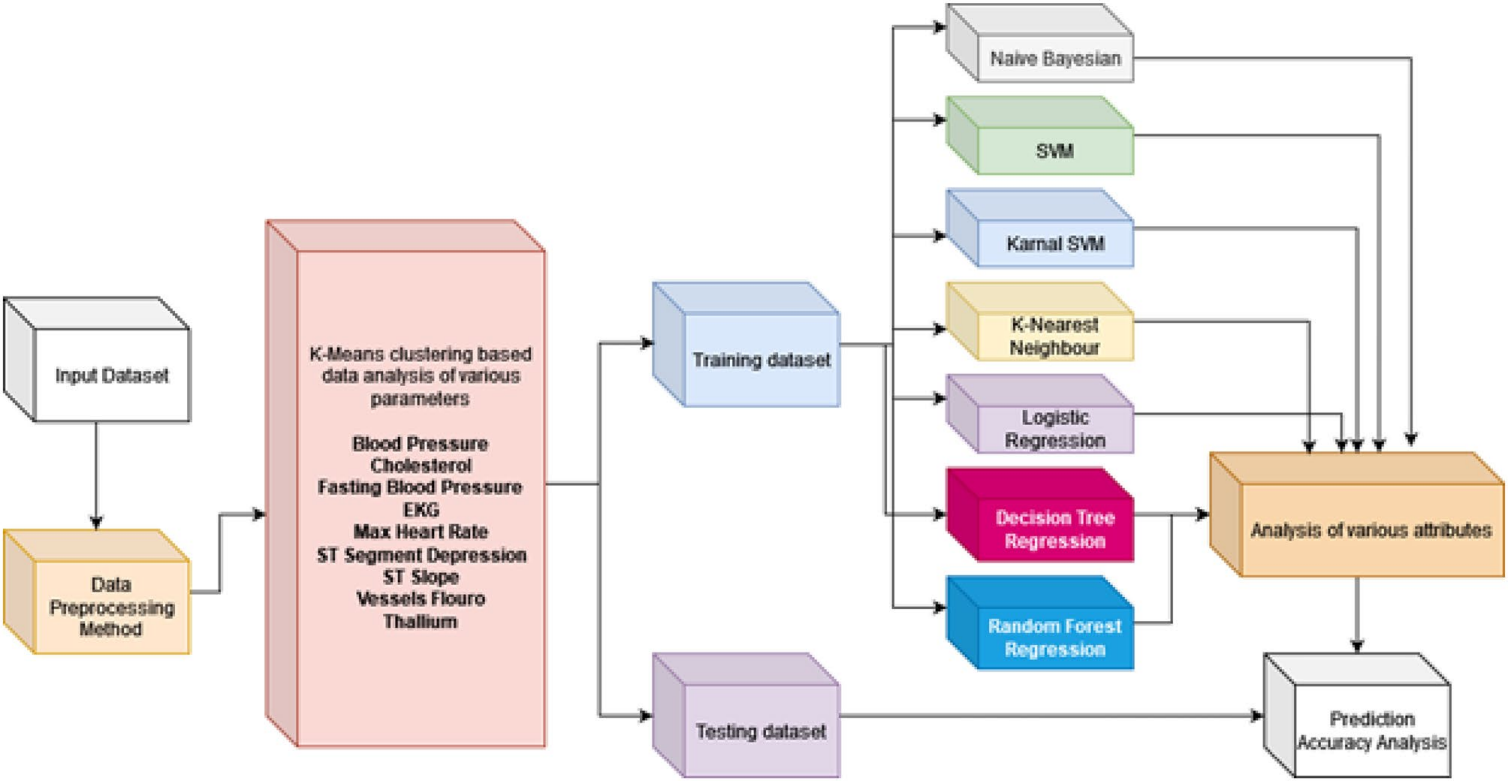
Proposed clustering cum regression analysis method.

The elbow method is utilized to estimate the optimal number of clusters. Despite its utility, this method has certain limitations, including subjectivity and the potential to oversimplify the data. To mitigate these limitations, the authors in^36^ have analyzed several methods to cross-validate, ensuring robustness. Of the many listed methods, we used a k-fold cross-validation approach to cross-validate the optimal cluster number to categorize the data into clusters. The clustering process utilizes Euclidean distance as the similarity measure, ensuring that data points are grouped based on their geometric proximity to the cluster centroids. To enhance the stability and efficiency of the algorithm, centroid initialization is performed using the k-means++ method, which strategically selects initial positions to minimize the risk of poor starting configurations and accelerate convergence. The data is initially segmented into clusters based on the optimal number of clusters identified. Following this clustering, the data is divided into training and testing datasets. The training dataset is analyzed using various regression algorithms to train the model for predicting heart disease. We evaluated the model’s performance using different regression methods, as illustrated in Fig. 2. For regression/classification, the dataset is divided into training and testing sets as given below:

3 $$\begin{aligned} D_{\text {train}} \cup D_{\text {test}} = D, \quad D_{\text {train}} \cap D_{\text {test}} = \emptyset \end{aligned}$$

This ensures that the model is trained and tested on mutually exclusive data subsets. Such a split is critical for unbiased performance evaluation and to prevent data leakage. Different regression models are used for the performance analysis. This model uses the sigmoid function to estimate the probability of heart disease. It is suitable for binary classification and interpretable in clinical settings.

4 $$\begin{aligned} f_{\text {LR}}(x) = \frac{1}{1 + e^{-w^T x}}, \quad \theta = w \end{aligned}$$

The SVM classifier finds the optimal hyperplane in a transformed feature space $\phi (x)$. It is effective in handling high-dimensional and non-linearly separable data. The model is defined below:

5 $$\begin{aligned} f_{\text {SVM}}(x) = \text {sign}(w^T \phi (x) + b) \end{aligned}$$

Another model is the Naïve Bayes classifier, which uses Bayes’ theorem. It assumes feature independence and is computationally efficient for real-time predictions.

6 $$\begin{aligned} P(y \mid x) \propto P(x \mid y) P(y) \end{aligned}$$

KNN assigns a class based on the majority label among the $k$ nearest neighbors. It is a non-parametric method that relies heavily on distance metrics.

7 $$\begin{aligned} f_{\text {KNN}}(x) = \text {majority}\left( y_j\right) , \quad j \in \text {NN}_k(x) \end{aligned}$$

The ensemble prediction of a Random Forest is defined below, which aggregates decisions from multiple trees. It reduces overfitting and improves classification robustness.

8 $$\begin{aligned} f_{\text {RF}}(x) = \text {majority}(f_1(x), f_2(x), \dots , f_T(x)) \end{aligned}$$

The data is classified based on the optimal number of clusters. After clustering, the data is further classified into a training and a testing dataset. The training dataset is again processed using various regression algorithms to train the model for predicting heart disease. Table 3 presents the hyperparameters configured for training the dataset across different machine learning models.

**Table 3:** Hyperparameters and their values for models.

**Model**	**Hyperparameter**	**Value**
Random Forest	n_estimators	200
	max_depth	10
	min_samples_split	2
	min_samples_leaf	1
	max_features	sqrt
SVM	kernel	rbf
	C	1.0
	gamma	scale
	class_weight	balanced
KNN	n_neighbors	5
	weights	distance
	metric	euclidean
	algorithm	auto

We analyzed the performance using various regression methods, as shown in Fig. 2. The analysis indicates that Random Forest outperforms the other methods. Consequently, Random Forest regression was chosen for the hybrid model due to its high accuracy, robustness against overfitting, and effective handling of high-dimensional data, making it a suitable complement to the clustering approach for enhancing prediction accuracy. Once the model is trained, the testing dataset is used to evaluate its prediction results. For the performance analysis of all models, the accuracy is considered a key parameter. The mathematical equation is defined below:

9 $$\begin{aligned} \text {Accuracy} = \frac{1}{|D_{\text {test}}|} \sum _{i=1}^{|D_{\text {test}}|} \mathbb {1}(\hat{y}_i = y_i) \end{aligned}$$

This metric calculates the ratio of correct predictions over the total number of test samples. Accuracy serves as a primary benchmark for model performance in binary classification tasks. The final predictive model returns the class with the highest posterior probability. This decision-making framework allows the incorporation of both statistical and learning-based classifiers. After theoretical data analysis, we processed the data using various pre-processing methods to minimize prediction errors.

10 $$\begin{aligned} f(x) = \arg \max _{y \in \{0, 1\}} \mathbb {P}(y \mid x; \theta ) \end{aligned}$$

The final regression cum clustering model is given below.

11 $$\begin{aligned} \hat{y}_{\text {new}} = \mathcal {F}_{k^*}(\textbf{x}_{\text {new}}) \quad \text {where } k^* = \arg \min _{k} \Vert \textbf{x}_{\text {new}} - \boldsymbol{\mu }_k\Vert ^2 \end{aligned}$$

Here, $\textbf{x}_{\text {new}}$ is first assigned to the nearest cluster $C_{k^*}$ using K-Means centroids $\boldsymbol{\mu }_k$. Then, the Random Forest model $\mathcal {F}_{k^*}$ trained on that cluster is used to predict $\hat{y}_{\text {new}}$. The model’s performance is assessed based on its learning capabilities during training and its prediction accuracy on the testing dataset. This evaluation is conducted using a confusion matrix, which analyzes True Positive and False Positive predictions. In our proposed approach, the dataset was split into two segments, i.e. 80% for training and 20% for testing. Data consistency is maintained using a prebuilt method for all regression methods. The hyperparameters are tuned using the prebuilt model in the scikit-learn library, i.e., the model-based optimization (MBO) method.

## Result analysis and discussion

In the present work, we have clustered the dataset of different heart-related parameters according to the patient’s age, recognizing that age is the most significant parameter for analysis. It has been found that almost all critical health parameters are affected by the patient’s age. Clustering played a pivotal role in improving the predictive performance of our model. By grouping features according to their similarity with respect to the age factor, clustering enabled the model to capture latent relationships among correlated variables and reduce redundancy in the input space. This structured grouping allowed the model to learn more coherent patterns and improve generalization. Comparative experiments demonstrated that models trained on clustered feature sets achieved higher prediction accuracy and lower error rates compared to those trained on ungrouped features. Thus, clustering served as an effective preprocessing step, strengthening the model’s ability to leverage age-related feature interactions and ultimately contributing to superior predictive outcomes.

**Fig. 3:**
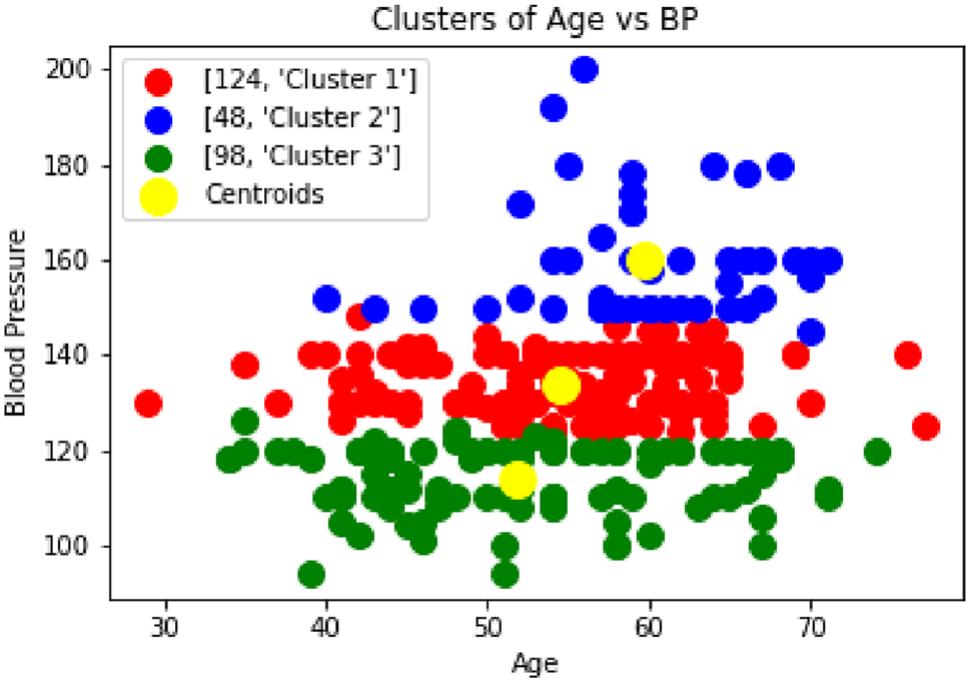
Clustering of Blood Pressure vs Age (3 clusters).

**Fig. 4:**
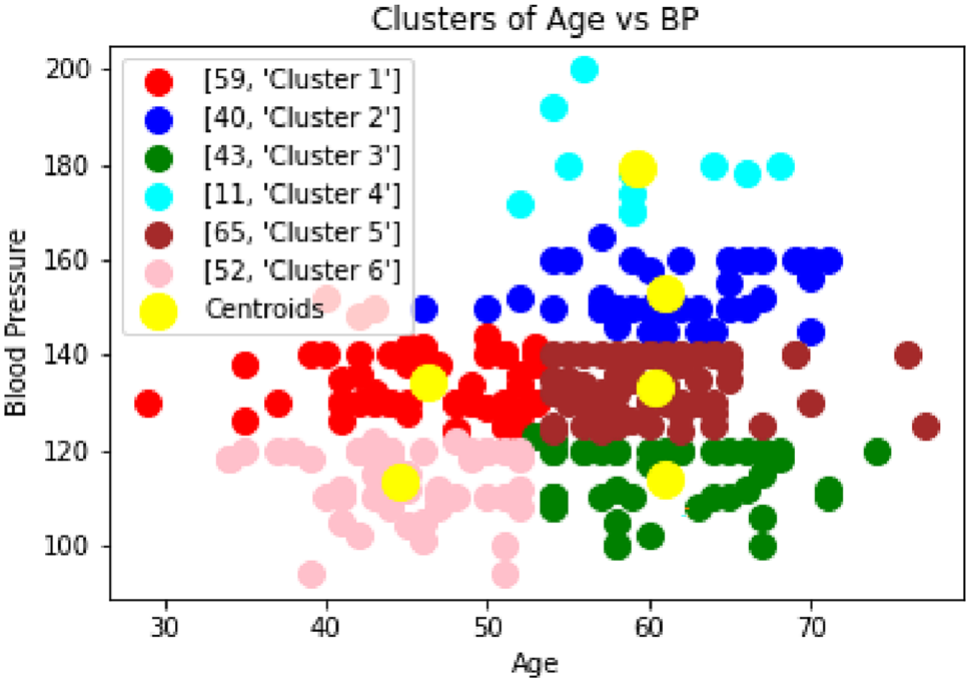
Clustering of Blood Pressure vs Age (6 clusters).

The actual prediction will be performed using a prediction algorithm. After prediction, patients will be classified into different categories based on their risk of heart disease. This method will be useful for the early prediction of heart disease. In the Fig. 3, the blood pressure is divided into 3 clusters. It is found that almost 14% of the patients can be categorized into risk zone i.e. those 48 patients categorized as ’Cluster 2’ whose Blood Pressure is 150 and above are at high risk of getting heart-related disease. Also, from Fig 4, it can be concluded that the 3% patients can be categorized into a highly risk zone i.e. those 11 patients categorized as ’Cluster 4’, whose Blood Pressure is 170 and above, are at high risk of getting heart-related disease in terms of the blood pressure.

**Fig. 5:**
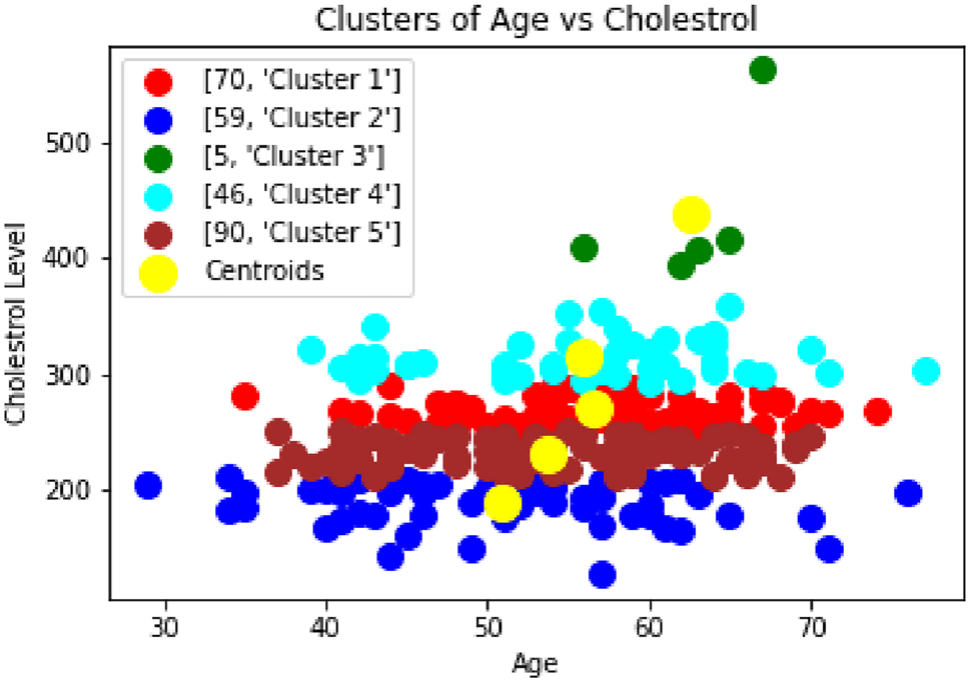
Clustering of Cholesterol Level vs Age.

**Fig. 6:**
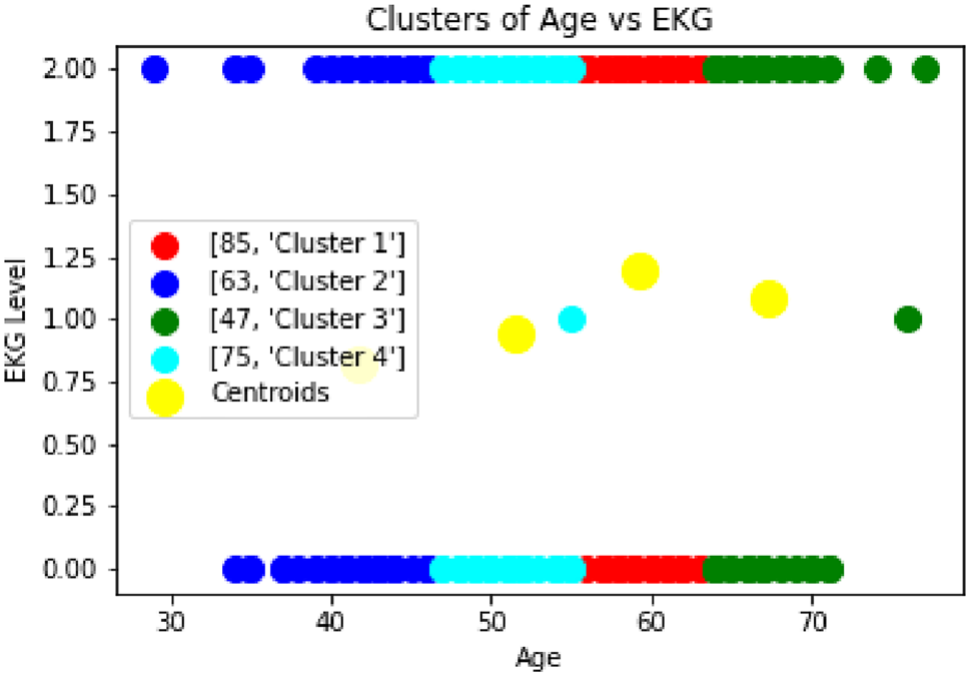
Clustering of EKG Level vs Age.

In Fig 5, we analyzed the cholesterol level of the patients. Based on the elbow method, it is found that five clusters are required to divide the patients. The result show that the 1–2% of the patients can be considered in the highly risk zone i.e. those 5 patients categorized as ’Cluster 3’ whose Cholestrol level is 400 and above are at high risk of getting heart-related disease and almost 17% of the patients are considered in the risk zone i.e. those 46 patients categorized as ’Cluster 4’ whose cholesterol level is 300 and above are at risk of getting heart-related disease. The EKG level also depends on the patient’s age, whether they are young or old. So it varies from time to time and depends on the other parameters. Hence, there is no relevance in considering this parameter alone to reach any conclusion. Therefore, from Fig 4, 5, and 6, it can be concluded that the patients with high risk level and second level of EKG can be considered as a high risk state. The study in^37^ identified a U-shaped correlation between HDL-C (High-Density Lipoprotein Cholesterol Levels) levels and mortality rates, both overall and cardiovascular-specific. This suggests that both very low and very high HDL-C levels are associated with increased mortality. These findings challenge the conventional belief that higher HDL-C levels are always advantageous, suggesting that extremely high HDL-C levels could be detrimental, especially for patients with pre-existing heart conditions.

**Fig. 7:**
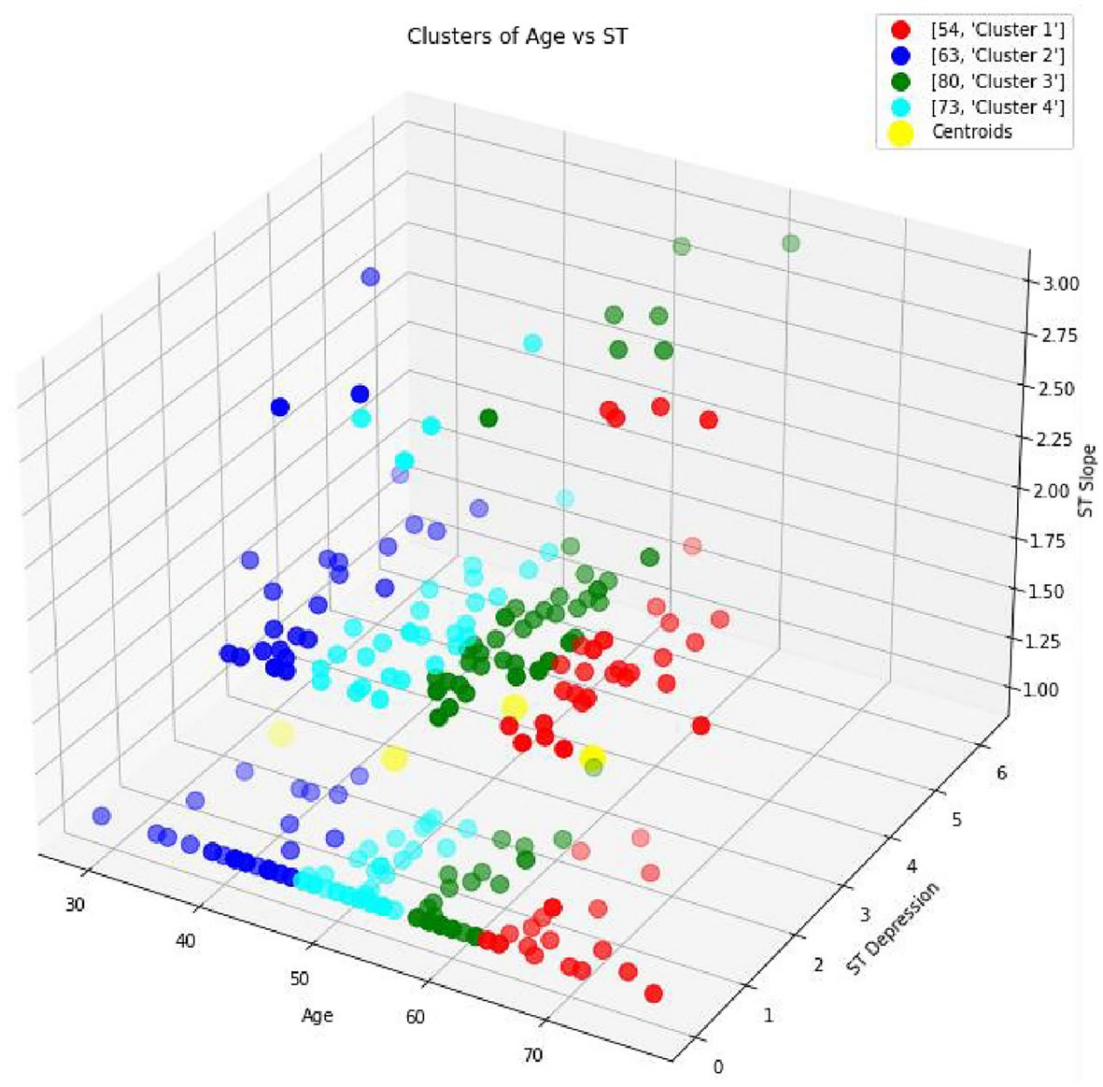
Clustering of ST Slope vs Age and ST Depression.

**Fig. 8:**
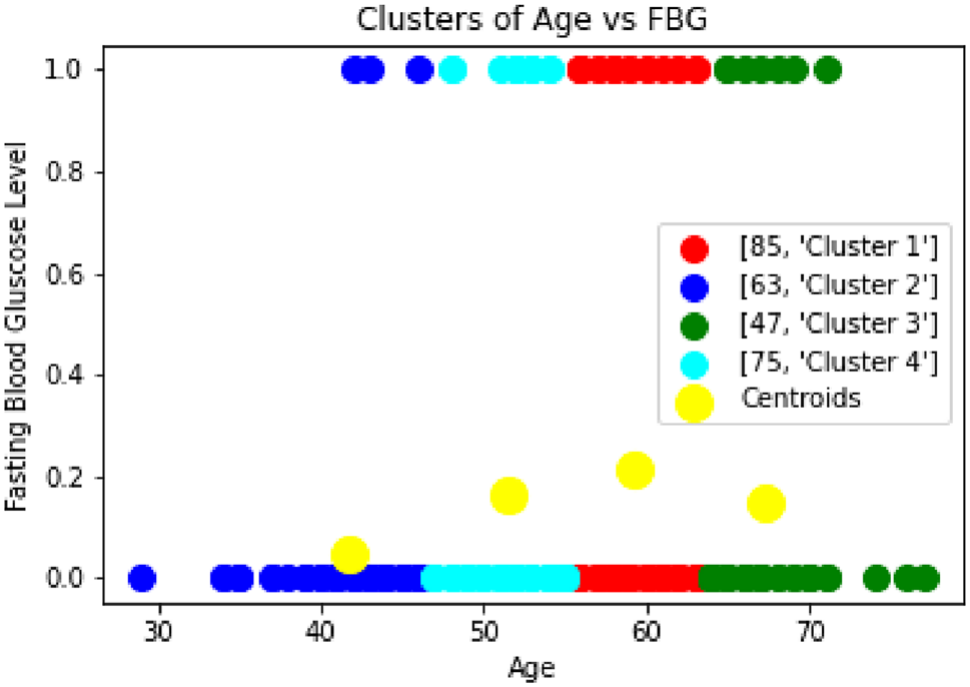
Clustering of Fasting Blood Glucose vs Age.

In the Fig 7, we have analyzed the ST slope and depression level using the ECG results of the patient. The results reveal that almost 14% of patients are considered in a highly risk zone and have a high chance of heart disease. The study^38^ explores the significance of ST depression in patients with coronary artery disease (CAD). It highlights that ST depression is a marker of myocardial ischemia and is associated with an increased risk of heart-related diseases, including myocardial infarction and sudden cardiac death. The findings underscore the importance of early detection and management of ST depression to improve patient outcomes. Similarly, Fig 8 shows that the 5–6 % patients are in age of 60–70 years and have risk level in terms of fasting blood sugar level.

**Fig. 9:**
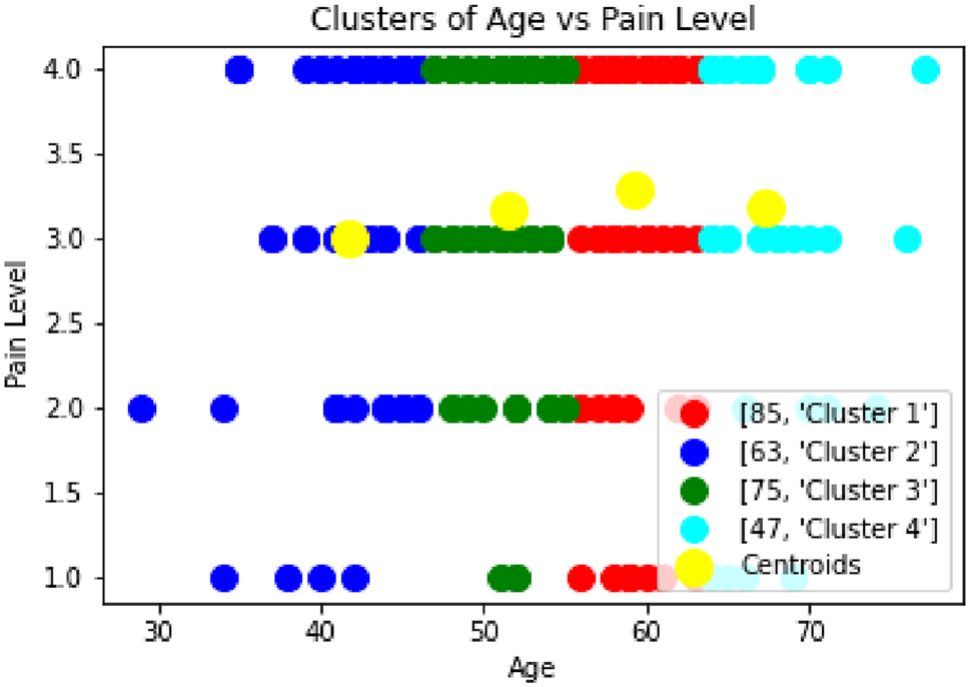
Clustering of Pain Level vs Age.

**Fig. 10:**
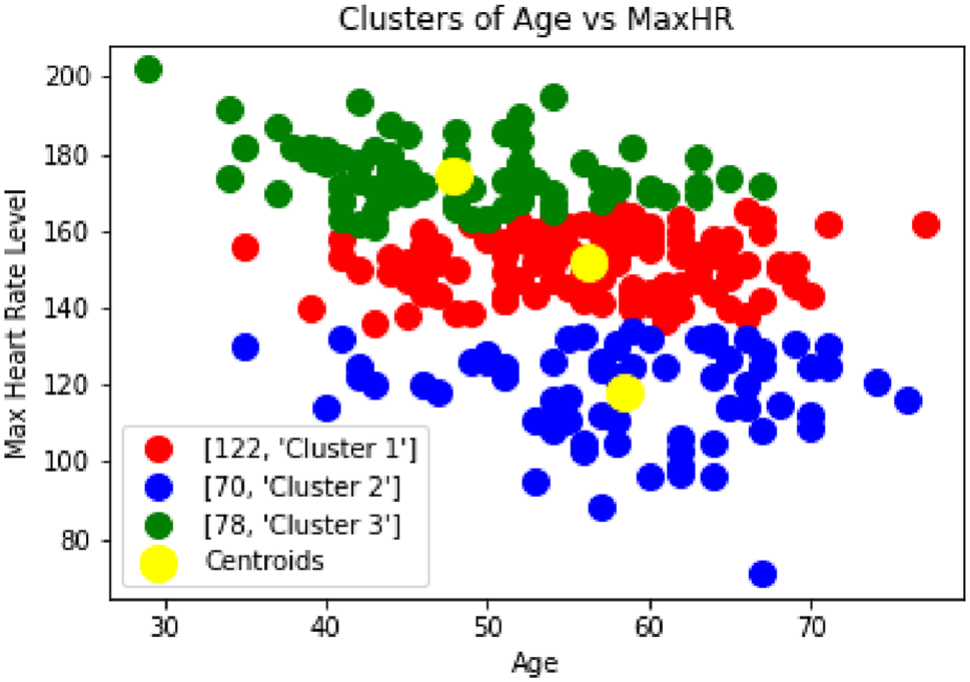
Clustering of Max Heart Rate Level vs Age.

The authors in^39^ established the reference values for heart rate variability (HRV) and examined its role in predicting clinical outcomes in individuals with heart failure and patients prone to heart-related diseases. It highlights that HRV markers are strong and independent predictors of survival, emphasizing their clinical relevance and potential for intervention in heart failure management. In the Fig. 9 and 10, the pain level and the maximum heart rate level are analyzed. The clustering of the pain level and critical heart level shows that approximately 20% of the patients are in the critical zone.

**Fig. 11:**
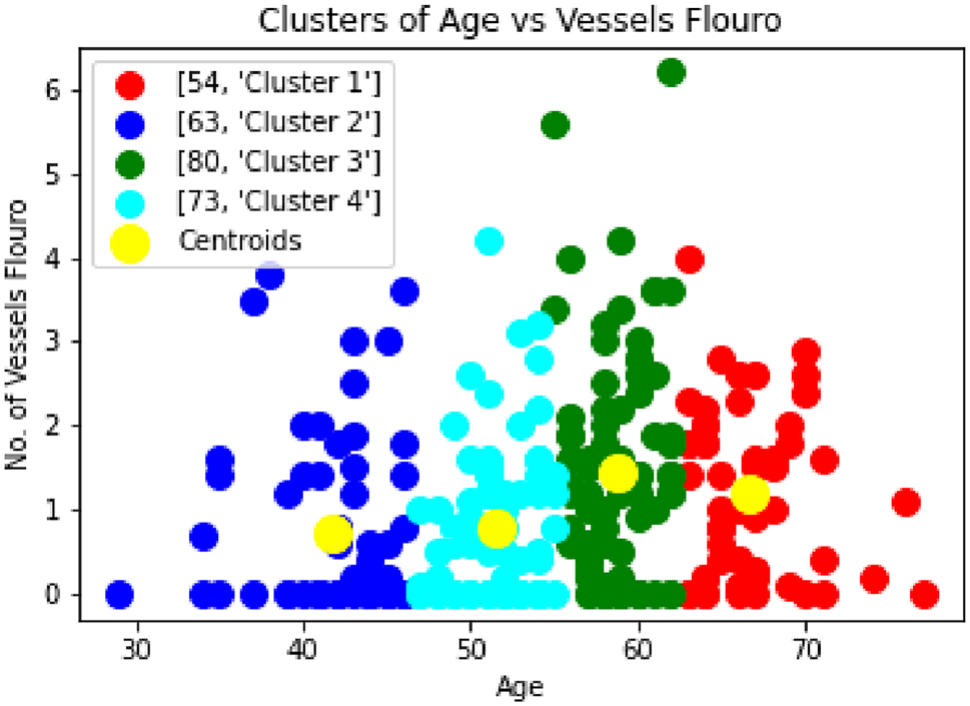
Clustering of Numbers of Vessels Flouro vs Age.

**Fig. 12:**
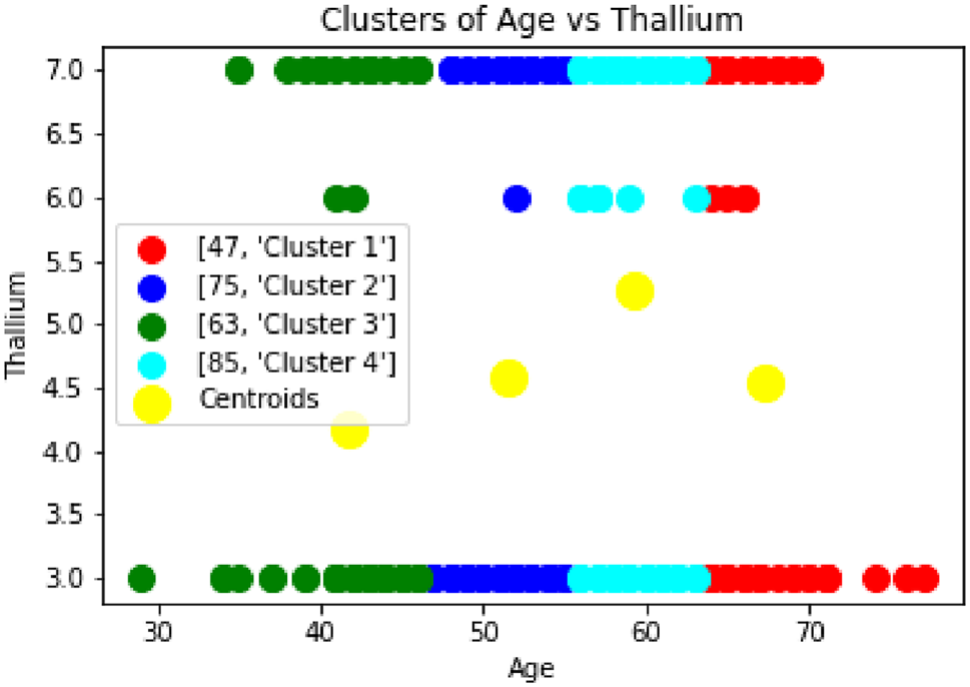
Clustering of Thallium vs Age.

The results shown in the Fig 11 and Fig 12 do not signify any important information related to the patient’s heart disease. The details are actually patient-specific and depend on other health parameters as well. The following information will help the doctor make an exact prediction of the heart disease. To predict heart disease, the effectiveness of various regression methods is thoroughly evaluated.

The results of the decision tree regression, K-Nearest Neighbor regression, Support Vector Machine, Kernel SVM, logistic regression, Naïve Bayes regression, and Random Forest regression methods are analyzed.

The accuracy and other parameter analysis is shown in the Table 4. In the comparative results, it is observed that the hybrid model (random forest regression with K-Means) outperforms the other regression methods (performs even better than the logistic regression method). The result shows that the accuracy of the random forest regression with K-Means algorithm is 91% which is better than the other reported works. Random Forest without clustering also performed well due to its ability to capture non-linear relationships and handle heterogeneous features, though slightly less effective than the clustered version. Decision Tree Regression achieved an accuracy of 76.47%, reflecting its interpretability but also its tendency to overfit on small datasets, as indicated by 8 false positives and 8 false negatives. K-Nearest Neighbor and Support Vector Machine showed moderate performance. KNN relies on local similarity, which can be sensitive to feature scaling and dimensionality. In contrast, SVM requires careful kernel tuning and is computationally intensive, limiting its practicality for real-time WBAN or IoMT deployments. Logistic Regression performed slightly better than KNN and SVM, but its assumption of linearity restricts its ability to model complex feature interactions. Naive Bayes had the lowest accuracy, likely due to its strong independence assumptions, which do not hold in this dataset, where features are correlated. The other neural network-based regression methods can also be analyzed; however, the computational time and complexity of the neural network-based prediction methods are higher than the above-discussed regression methods. The proposed method gives higher prediction accuracy without compromising the computational time.

The results show that the training and testing times for all methods are comparable, ranging from milliseconds to seconds, with only slight differences from existing techniques.

**Table 4:** Evaluation Metrics for Heart Disease Prediction Models.

Model	TP	FP	FN	TN	Accuracy	Recall	Specificity	F1 Score	ROC–AUC
Decision Tree Regression	32	8	8	20	0.7647	0.8000	0.7143	0.7619	0.7624
K-Nearest Neighbor	32	8	7	21	0.7794	0.8205	0.7241	0.7742	0.8896
Support Vector Machine	32	8	6	22	0.7941	0.8421	0.7333	0.7843	0.9049
Kernel SVM	32	8	7	21	0.7794	0.8205	0.7241	0.7742	0.9048
Logistic Regression	32	8	6	22	0.7941	0.8421	0.7333	0.7843	0.9019
Naïve Bayes	32	8	7	21	0.7794	0.8205	0.7241	0.7742	0.9062
Random Forest Regression	37	8	9	19	0.8235	0.8043	0.7037	0.7989	0.9167
Random Forest with K-Means Analysis	39	1	5	23	0.9100	0.8864	0.9583	0.8977	0.9155

**Note:** TP=True Positive, FP=False Positive, FN=False Negative, TN=True Negative, ROC=Receiver Operating Characteristic, AUC=Area Under the (ROC) Curve.

To statistically validate whether the proposed Random Forest with K-Means model provides a significant improvement over other classifiers, 95% confidence intervals for accuracy were computed for all models as shown in Table 5. The proposed hybrid model achieved an accuracy of 91.18%, with a confidence interval of 0.844–0.979, which does not overlap with the confidence intervals of any baseline classifier (ranging from 0.664–0.890). The non-overlapping confidence intervals confirm that the improvement is statistically significant at the 95% confidence level, demonstrating that the performance gain is attributable to the proposed methodology rather than random variation.

**Table 5:** Statistical Significance Testing Using 95% Confidence Intervals.

Model	Accuracy	95% CI (Lower)	95% CI (Upper)
Decision Tree Regression	0.7647	0.6639	0.8655
K-Nearest Neighbor	0.7794	0.6809	0.8780
Support Vector Machine	0.7941	0.6980	0.8902
Kernel SVM	0.7794	0.6809	0.8780
Logistic Regression	0.7941	0.6980	0.8902
Naïve Bayes	0.7794	0.6809	0.8780
Random Forest Regression	0.7671	0.6702	0.8641
**Random Forest + K-Means**	**0.9118**	**0.8443**	**0.9792**

Notably, the proposed hybrid clustering-cum-regression model outperforms current regression models in performance. In heart disease prediction applications, accuracy is paramount, making time complexity, training duration, and computational demands secondary considerations. Additionally, the random forest algorithm and the proposed method exhibit higher time complexity, training duration, and computational demands compared to other algorithms.

The dataset, sourced from the UCI repository, is relatively small and may not fully represent real-world clinical populations. To mitigate the risks of overfitting, we employed cross-validation and regularization across all models. The ensemble nature of Random Forest further mitigates overfitting by averaging multiple decision trees, reducing variance compared to a single tree.

This study acknowledges ethical considerations regarding machine-learning-based medical decision systems, including transparency, patient consent, and potential biases. Furthermore, predicting heart disease solely from structured features has inherent limitations, as it may overlook unstructured clinical data, imaging, and patient-reported outcomes that could improve diagnostic accuracy.

## Conclusion

In this article, a K-means-based hybrid clustering-cum-regression method is proposed for testing and training datasets to facilitate efficient disease prediction. The proposed method improves the accuracy of the existing method without compromising the time complexity. The database is divided into 80% of training and 20% of testing datasets. Furthermore, the data analysis is performed for the 12 feature parameters, which are useful for precise heart disease detection. The parameters are also analyzed using various regression methods for the relevant feature parameters after processing K-means clustering for the training dataset. The K-Means algorithm is used to analyze the relevance of a parameter within a particular range. Additionally, the confusion matrix is analyzed to calculate the accuracy for the performance comparison of heart disease detection. The proposed hybrid model achieves 91% prediction accuracy by combining random forest with K-means analysis. The results prove the superiority of the proposed hybrid model. In summary, the proposed model shows promising capability in predicting heart disease within the WBAN healthcare system. Nevertheless, its effectiveness is influenced by the dataset’s characteristics and potential biases, and the reliance on structured features may limit generalizability across diverse clinical settings.

For future work, integrating advanced ensemble learning strategies or deep learning architectures with the clustering stage could significantly enhance predictive performance, potentially surpassing the current accuracy achieved with the random forest approach. Such integration would enable the model to capture complex, non-linear patterns in the data while ensuring scalability and adaptability for large and dynamically evolving datasets.

## Data Availability

The datasets used in this study are publicly available and can be accessed through the below-given link/platform. University of California Irvine’s Machine Learning Repository at https://archive.ics.uci.edu/ml/datasets/Heart+Disease.
